# Supplementary material for: The Evolution and Characterization of the RNA Interference Pathways in Lophotrochozoa
Source: Genome Biol Evol. 2024 May 7;16(5):evae098. doi: 10.1093/gbe/evae098 (PMC11114477; doi:10.1093/gbe/evae098)
Supplement: evae098_Supplementary_Data [file evae098_supplementary_data.zip › Supplementary_materials.zip!/selected_miRNAs/NoGe_selected_miRNAs/mirdeep_pdf_files/NMRB01000279.1_Notospermus_geniculatus_strain_Ushimado_scaffold279,_whole_genome_shotgun_sequence_9062.pdf]

Provisional ID	:	NMRB01000279.1_Notospermus_geniculatus_strain_Ushimado_scaffold279,_whole_genome_shotgun_sequence_9062
Score total	:	0
Score for star read(s)	:	-1.3
Score for read counts	:	0
Score for mfe	:	1.9
Score for randfold	:	0
Score for cons. seed	:	-0.6
Total read count	:	57
Mature read count	:	49
Loop read count	:	0
Star read count	:	8

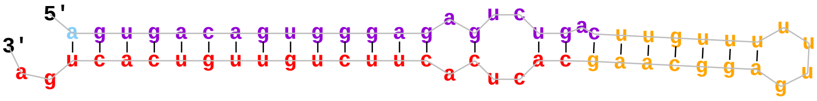

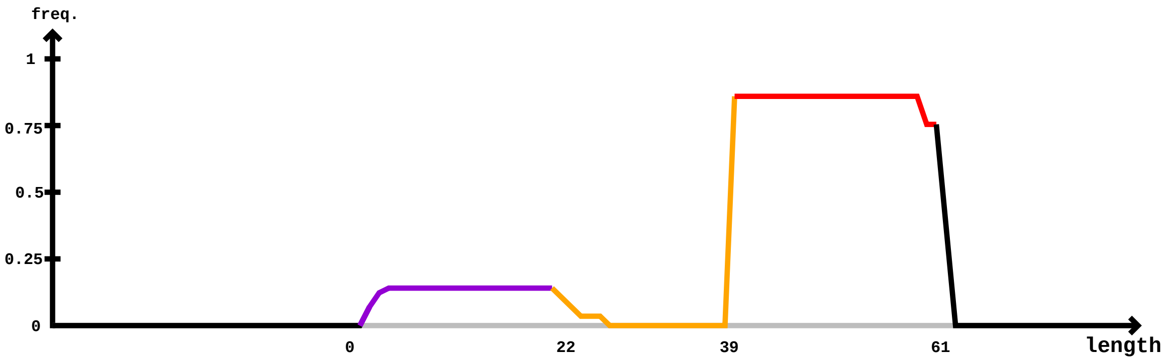

Star Mature

```
5'- gaagccgggcgcagcgaugcgucagccgucagugacagugggagagucugacuuguuuuuugaggcaagcacucacuucuguugucacugaugucuggagagaucgaacaa  -3'   obs
    gaagccgggcgcagcgaugcgucagccgucagugacagugggagagucugacuuguuuuuugaggcaagcacucacuucuguugucacugaugucuggagagaucgaacaa         exp
    ...((..((((((....)))))).))(((((((((((((((((((.(..((.(((((((....)))))))))..).))))))))))))))))))(((....)))........   reads   mm   sample
    ...............................gugacagugggagagucugac...........................................................   3       0    Fm3
    ................................ugacagugggagagucugacu...........................................................   3       0    Fm3
    .................................gacagugggagagucugacuugu........................................................   1       0    Fm3
    ......................................................................cacucacuucuguugucacugU....................   15      1    Fm3
    ......................................................................cacucacuucuguugucacCga....................   5       1    Fm3
    ......................................................................cacucacuucuguugucacuga....................   5       0    Fm3
    ......................................................................cacucacuucuguugucacugUu...................   1       1    Fm3

    ......................................................................cacucacuucuguugucacugU....................   1       1    Fm1

    ...............................gugacagugggagagucugacuugu........................................................   1       0    Mm2
    ......................................................................cacucacuucuguugucacCga....................   2       1    Mm2

    ......................................................................cacucacuucuguugucacu......................   6       0    Mm1
    ......................................................................cacucacuucuguugucacugU....................   14      1    Mm1
```
